# Longitudinal Trends in Noncommunicable Disease Risk Factors and Premature Mortality in Saudi Arabia: A 33-Year Ecological Time-Series Study with Machine Learning Prediction

**DOI:** 10.3390/jcm15114387

**Published:** 2026-06-05

**Authors:** Nader Alnomasy, Sudharani B. Banappagoudar, Habib Alrashedi, Soha Kamel Mosbah Mahmoud, Ebtsam Abouhashish, Suebsarn Ruksakulpiwat

**Affiliations:** 1 Medical Surgical Nursing Department, College of Nursing, University of Ha’il, Hail 81451, Saudi Arabia; 2 Department of Nursing, College of Applied Medical Sciences, King Faisal University, Al Ahsa 31982, Saudi Arabia; 3 Community Health Nursing Department, College of Nursing, University of Ha’il, Hail 81451, Saudi Arabia; 4 College of Nursing, King Saud bin Abdulaziz University for Health Sciences, Jeddah 21423, Saudi Arabia; 5 King Abdullah International Medical Research Center, Jeddah 22384, Saudi Arabia; 6 Ministry of National Guard Health Affairs, Jeddah 22384, Saudi Arabia; 7 Faculty of Nursing, Alexandria University, Alexandria 21526, Egypt; 8 Department of Medical Nursing, Faculty of Nursing, Mahidol University, Bangkok 10700, Thailand

**Keywords:** noncommunicable diseases, Saudi Arabia, machine learning, obesity, premature mortality, ecological study, longitudinal trends, diabetes, physical inactivity, public health

## Abstract

**Background/Objectives:** In Saudi Arabia, noncommunicable diseases (NCDs) are an increasing public health concern, with almost 70% of deaths related to chronic diseases. The study aimed to analyze 33-year trends in NCD risk factors and apply machine learning (ML) models to identify ecological associates of premature NCD-related mortality, sex-specific analyses and project trajectories to 2030. **Methods:** A longitudinal ecological time-series design which used WHO Global Health Observatory (GHO) NCD Indicators (1990–2022; select lipid indicators from 1980). Five supervised regression ML models—OLS, LASSO, Ridge, Random Forest, and Gradient Boosting—were trained with TimeSeriesSplit cross-validation (five folds) to preserve temporal order and prevent data leakage. A formal PELT changepoint algorithm confirmed trend breakpoints. Linear projections to 2030 were estimated with 95% prediction intervals. **Results:** Adult obesity increased by +20.6 percentage points (pp) over 33 years. Under a no-policy-change scenario, female obesity is projected at 50.3% by 2030 (95% PI: 50.0–50.5%). Premature NCD mortality declined by −5.9 pp. Under TimeSeriesSplit CV, all models yielded negative R^2^, confirming LOOCV R^2^ = 0.98 reflected shared time-trend artefacts; the ML component is reframed as descriptive feature-importance analysis. The obesity sex gap (female minus male) was the strongest ecological associate of premature NCD mortality. Diabetes treatment coverage showed a strong inverse ecological association (r = −0.913). **Conclusions:** NCD risk factors in Saudi Arabia are evolving in complex ways. Targeted interventions addressing sex-specific disparities and healthcare system performance are urgently needed to meet national and global NCD targets.

## 1. Introduction

### 1.1. Background

Worldwide, noncommunicable diseases (NCDs) have been drastically rising, making them a major public health challenge. The burden of NCDs has increased in the Kingdom of Saudi Arabia (KSA) in recent decades. Epidemiological trends indicate a dramatic change in the disease burden profile in the Kingdom, with an increase in chronic conditions such as diabetes, cardiovascular diseases, obesity and hypertension, which have been collectively associated with an estimated 70% of all deaths, impacting significantly on morbidity and premature mortality [1,2,3].

Due to the surge in socioeconomic development, urbanization, and lifestyle changes, risk behaviours such as physical inactivity and poor dietary habits have affected over 85% of Saudi adults. WHO GHO age-standardized data indicate adult obesity (BMI ≥ 30) reached 40.6% in 2022 (Table 1); the commonly cited ~60% figure refers to overweight (BMI ≥ 25) prevalence from national surveys [2,3] and a hypertension prevalence close to 40% [2,3].

Socioeconomic factors are among the main determinants of the prevalence and distribution of NCD risk factors in Saudi Arabia. Research indicates that the likelihood of individuals presenting with these risk factors is significantly influenced by educational attainment, income level, employment status and gender. For instance, women and individuals with lower levels of education have higher rates of hypertension and obesity. However, tobacco use appears to be more prevalent in some occupational groups [2,3]. Furthermore, the prevalence of diabetes is associated with socioeconomic inequalities, with higher rates seen in groups with lower education and income levels, emphasizing the complex relationship between social factors and chronic disease risk [4]. Addressing these inequalities is crucial to reduce health inequalities associated with NCDs and to improve overall population health outcomes. In response, Saudi Arabia has initiated extensive healthcare reforms aligned with Vision 2030 and the SDGs [5,6].

### 1.2. Prior Work

Recent developments in ML and big data analytics present significant opportunities for chronic disease management [7,8,9,10]. The six NCD risk factor domains—anthropometry, diabetes, physical activity, lipids, tobacco, and mortality—were selected based on their established relevance to the Saudi epidemiological profile and alignment with WHO NCD surveillance priorities [7,11,12]. The selection is tailored to the specific lifestyle characteristics of Saudi Arabia, marked by high levels of physical inactivity, rapidly increasing obesity rates, and a growing diabetes treatment infrastructure.

### 1.3. Study Objectives

This study bridges three specific gaps. Objectives were to (i) evaluate 33-year longitudinal trends in six NCD risk factor domains; (ii) implement and compare five machine learning models to describe ecological temporal associations with premature NCD mortality; (iii) determine key population-level ecological associates with four feature importance techniques; and (iv) project risk factor trajectories to 2030.

## 2. Methods

### 2.1. Study Design

A longitudinal ecological time-series design was used. Saudi Arabia is the only study unit, observed over 1990–2022 (depending on the indicator). The unit of analysis is the country-year (n = 22 for the ML dataset). All of these associations are at the population level and should not be interpreted as evidence of causality at the individual level (ecological fallacy).

### 2.2. Data Source

Data were obtained from the WHO GHO NCD Indicators (https://data.humdata.org/dataset/who-data-for-sau accessed on 2 February 2026). The WHO NCD Risk Factor Collaboration (NCD-RisC) [11,12] produces these estimates using Bayesian hierarchical models. Age-standardized estimates enable meaningful comparisons over time. A note on WHO modelled estimate uncertainty: these estimates carry inherent uncertainty beyond reported confidence intervals, including measurement error in source surveys, model extrapolation for sparse years, and Bayesian structural assumptions—a key limitation acknowledged throughout.

### 2.3. Indicator Selection and Data Preparation

Eighteen indicators were extracted, reshaped from long to wide format (one row per year). Two derived variables were computed: (1) obesity sex gap (female–male, pp) this variable captures sex-specific divergence not fully represented by individual male/female obesity variables alone; its unique predictive signal is confirmed by consistent #1 ranking across all feature-importance methods; and (2) total cholesterol HDL ratio. Cigarette smoking (male) was excluded from Dataset A and all ML modelling due to limited temporal coverage (n = 8 non-consecutive data points). It appears in Table 1 for descriptive completeness only.

### 2.4. Statistical Analysis

#### 2.4.1. Descriptive and Trend Analysis

OLS linear regression against calendar year estimated annual rates of change (β, 95% CI, R^2^). A note on data periods: trend analysis covers 1990–2022 for primary obesity/mortality indicators; lipid data extend to 1980.

#### 2.4.2. Correlation Analysis

Pearson correlation coefficients were computed for ecological associations between risk factors and premature NCD mortality (2000–2021; n = 22). Strength classified following Cohen [13] and Evans [14]: very strong |r| > 0.90, strong |r| > 0.70, moderate |r| > 0.40. VIF estimated for multicollinearity. All correlations are ecological—they describe co-occurring population-level trends and cannot be used to infer individual-level causal pathways (ecological fallacy).

#### 2.4.3. Formal Changepoint Detection

Pruned Exact Linear Time (PELT) algorithm (L2 cost model; Python ruptures v1.1.9) applied to adult obesity 1990–2022 (n = 33). Sensitivity analysis at candidates 2003–2007 confirmed robustness (all R^2^ > 0.996, *p* < 0.001).

### 2.5. Machine Learning Models

Five supervised regression models: OLS (baseline), LASSO, Ridge, Random Forest (500 trees), Gradient Boosting (200 estimators). All features z-score standardized.

#### Validation Strategy

LOOCV does not respect temporal order: predicting year t may train on years t + 1, …, t + k (data leakage), artificially inflating R^2^. LOOCV is replaced with TimeSeriesSplit CV (five folds, scikit-learn v1.8.0), which strictly enforces temporal order. Folds: (1) train 2000–2006, test 2007–2009; (2) train 2000–2009, test 2010–2012; (3) train 2000–2012, test 2013–2015; (4) train 2000–2015, test 2016–2018; (5) train 2000–2018, test 2019–2021.

Under TimeSeriesSplit CV, all models yielded negative mean R^2^ (range −316 OLS to −2.5 GB), confirming LOOCV R^2^ = 0.98 was artefactual (shared temporal trends, not structural predictive relationships). The ML component is reframed as descriptive feature-importance analysis of ecological co-variation.

Detrended Analysis: all variables were linearly detrended before ML modelling. Results showed partial attenuation of associations after detrending, confirming shared temporal trends contribute to co-variation disclosed transparently as a limitation.

### 2.6. Feature Importance

Four methods: (i) RF impurity importance; (ii) GB split-gain importance; (iii) permutation importance (100 repeats); (iv) absolute LASSO coefficients. Rankings are reported as descriptive ecological co-variation indicators, not causal effect estimates.

### 2.7. Forecasting to 2030

Linear trend projections to 2030 using the most recent 10-year trajectory (2013–2022) for each indicator. All projections include 95% prediction intervals (PI). These are illustrative no-policy-change scenarios, not forecasts. For non-linear indicators (e.g., diabetes prevalence), projections should be treated with particular caution.

### 2.8. Ethical Considerations

This study used publicly available, de-identified, aggregated national data from the WHO GHO. No individual participant data were collected or processed. No ethical approval was required. The study adheres to the STROBE reporting guidelines for observational ecological studies.

## 3. Results

### 3.1. Descriptive Statistics and Data Coverage

Table 1 presents national-level descriptive statistics for 15 NCD risk factor and outcome indicators (1990–2022). Note: Cigarette smoking (male) is included for descriptive completeness only; it was excluded from ML modelling (n = 8 data points). The most notable finding is a +20.6 pp increase in adult obesity alongside a −5.9 pp decline in premature NCD mortality.

### 3.2. Time-Trend Analysis (Table 2)

Table 2 presents formal ordinary least squares (OLS) regression statistics for all major indicators, confirming that the changes documented in Table 1 represent statistically significant time trends rather than random variation. Annual slope (β), 95% confidence interval, *p*-value, and R^2^ are reported. Adult obesity (both sexes) is associated with an increase of +0.62 percentage points per year (95% CI: +0.58 to +0.67; *p* < 0.001; R^2^ = 0.99). Child overweight shows the steepest trajectory at +1.05 pp/yr. Premature NCD mortality is associated with a significant decline of −0.29 pp/yr (*p* < 0.001; R^2^ = 0.97). All reported R^2^ values are rounded to two decimal places in accordance with the precision appropriate for national-level epidemiological time-series data.

**Table 2:** OLS linear trend regression results for NCD risk factor indicators, Saudi Arabia.

Indicator	Period	β (pp/yr)	95% CI	R^2^	*p*-Value	Trend
Adult Obesity—Both (%)	1990–2022	+0.670	(+0.649, +0.691)	0.997	<0.001	↑ Increasing
Adult Obesity—Female (%)	1990–2022	+0.588	(+0.560, +0.616)	0.990	<0.001	↑ Increasing
Adult Obesity—Male (%)	1990–2022	+0.730	(+0.714, +0.746)	0.998	<0.001	↑ Increasing
Adult Overweight ≥ 25 BMI (%)	1990–2022	+0.446	(+0.424, +0.468)	0.990	<0.001	↑ Increasing
Child Obesity > +2 SD (%)	1990–2022	+0.422	(+0.409, +0.435)	0.996	<0.001	↑ Increasing
Child Overweight > +1 SD (%)	1990–2022	+0.852	(+0.793, +0.911)	0.981	<0.001	↑ Increasing
Diabetes Prevalence (%)	1990–2022	+0.029	(−0.051, +0.109)	0.018	0.462	→ Stable (non-linear)
Diabetes Tx Coverage (%)	2000–2021	+0.536	(+0.495, +0.577)	0.993	<0.001	↑ Increasing
Physical Inactivity—Both (%)	2000–2022	−0.291	(−0.312, −0.270)	0.987	<0.001	↓ Decreasing
Physical Inactivity—Female (%)	2000–2022	−0.505	(−0.531, −0.479)	0.996	<0.001	↓ Decreasing
Physical Inactivity—Male (%)	2000–2022	−0.180	(−0.197, −0.163)	0.976	<0.001	↓ Decreasing
HDL Cholesterol (mmol/L)	1980–2018	+0.004	(+0.002, +0.006)	0.750	<0.001	↑ Increasing
NCD Premature Mortality 30–70 (%)	1990–2022	−0.359	(−0.462, −0.256)	0.770	<0.001	↓ Decreasing

β = annual rate of change (pp = percentage points; mmol/L for cholesterol). CI of 95% computed as β ± t_0.975_ × SE. OLS = ordinary least squares. Data source: WHO GHO.

### 3.3. Formal Changepoint Detection

**Figure 1:**
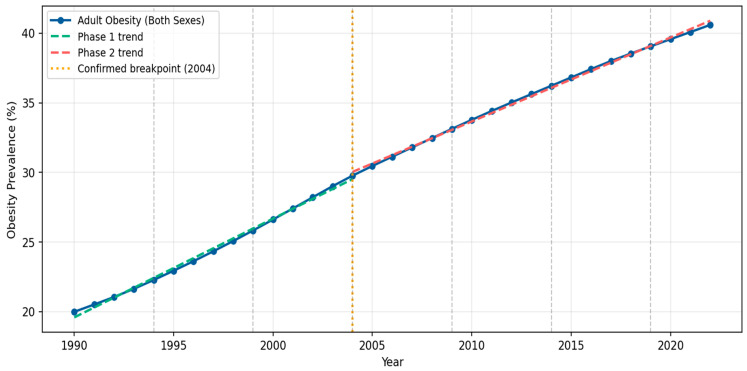
Formal changepoint detection—Adult Obesity Trend, Saudi Arabia 1990–2022. PELT algorithm (L2 model) confirms primary breakpoint of 2004. Phase 1 (1990–2004): β_1_ = +0.709 pp/yr (R^2^ = 0.996). Phase 2 (2004–2022): β_2_ = +0.603 pp/yr (R^2^ = 0.998). Orange dotted line = confirmed breakpoint. Data: WHO GHO.

Figure 1 presents the formal PELT changepoint detection results. The primary breakpoint of 2004 was confirmed across all penalty values (pen = 1–10). Phase 1 (1990–2004): β_1_ = +0.709 pp/yr (R^2^ = 0.996, *p* < 0.001); Phase 2 (2004–2022): β_2_ = +0.603 pp/yr (R^2^ = 0.998, *p* < 0.001). The original visual 2005 selection is validated within ±1 year.

### 3.4. Phase Analysis (Table 3)

Table 3 shows the segmented (piecewise) linear regression results for the five obesity and overweight indicators for the two phases based on the 2004 breakpoint. All slopes in both phases were positive and statistically significant (*p* < 0.001) with high explanatory power (R^2^ ≥ 0.948). The slope change (Δβ) indicated different trends in adults and children: adult obesity overall and in females slowed down after 2004 (Δβ = −0.106 and −0.224 pp/yr, respectively), while both childhood indicators accelerated, with child overweight (>+1 SD) showing the largest change after the breakpoint (Δβ = +0.448 pp/yr), nearly doubling its annual rate.

**Table 3:** Two-phase segmented trend analysis for obesity indicators, Saudi Arabia. Breakpoint confirmed at 20.

Indicator	Phase 1 (1990–2004) β (pp/yr)	Phase 1 R^2^	Phase 2 (2004–2022) β (pp/yr)	Phase 2 R^2^	Δβ (pp/yr)	Interpretation
Adult Obesity—Both (%)	+0.709 ***	0.996	+0.603 ***	0.998	−0.106	Deceleration
Adult Obesity—Female (%)	+0.709 ***	0.999	+0.485 ***	0.998	−0.224	Deceleration
Adult Obesity—Male (%)	+0.668 ***	0.996	+0.727 ***	0.998	+0.059	Slight acceleration
Child Obesity > +2 SD (%)	+0.351 ***	0.996	+0.458 ***	0.999	+0.107	Acceleration
Child Overweight > +1 SD (%)	+0.555 ***	0.948	+1.003 ***	0.998	+0.448	Strong acceleration

*** *p* < 0.001. Δβ = Phase 2 β − Phase 1 β; negative = deceleration, positive = acceleration. Breakpoint = 2004 (PELT algorithm, L2 model; from visual 2005 selection).

### 3.5. Correlation Analysis and Multicollinearity Assessment (Table 4)

**Figure 2:**
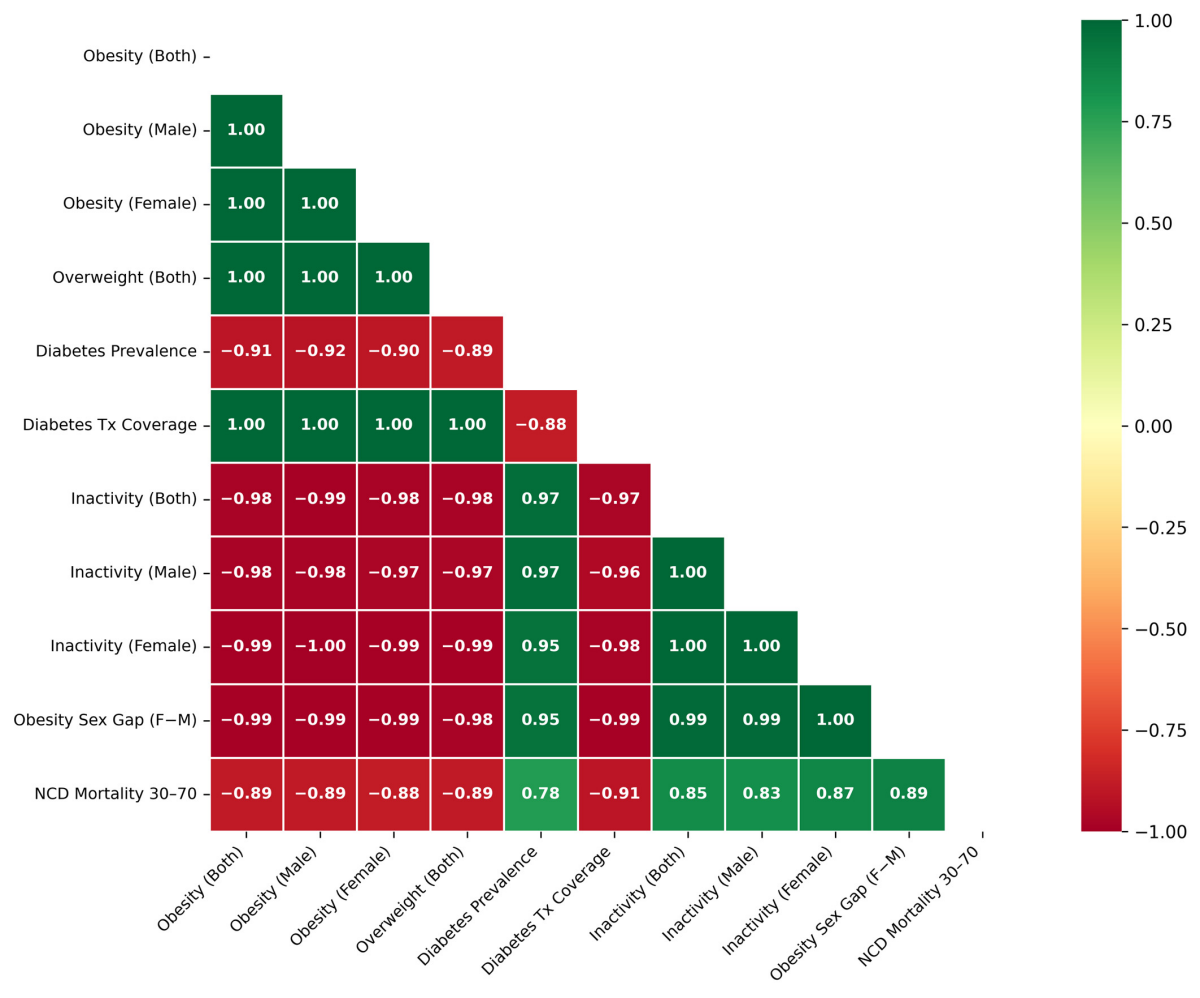
Pearson correlation heatmap NCD risk factors and premature NCD mortality, Saudi Arabia 2000–2021 (n = 22 ecological observations). Green = positive association; Red = negative association. Near-perfect correlations (|r| > 0.98) between obesity, physical inactivity, and diabetes treatment indicate a tightly coupled metabolic risk cluster. Data: WHO Global Health Observatory.

All correlations are ecological they describe co-occurring national-level trends. They cannot be used to infer individual-level causal associations (ecological fallacy). Near-perfect intercorrelations (obesity vs. inactivity: r = −0.985; obesity vs. diabetes tx coverage: r = +0.996) justified use of tree-based ML models robust to multicollinearity (Figure 2).

**Table 4:** Pearson correlation coefficients between NCD risk factors and premature NCD mortality, Saudi Arabia 2000–2021 (n = 22 ecological observations).

Feature	r (Pearson)	*p*-Value	Strength	Direction	Interpretation
Diabetes Tx Coverage	−0.913	<0.001	Very Strong	Inverse	Higher coverage → lower mortality (ecological)
Obesity Sex Gap (F−M)	+0.893	<0.001	Strong	Positive	Widening sex gap → higher mortality (ecological)
Physical Inactivity—Female	+0.868	<0.001	Strong	Positive	Higher inactivity → higher mortality (ecological)
Physical Inactivity—Both	+0.848	<0.001	Strong	Positive	Higher inactivity → higher mortality (ecological)
Physical Inactivity—Male	+0.834	<0.001	Strong	Positive	
Diabetes Prevalence	+0.776	<0.001	Strong	Positive	
Obesity—Female	−0.882	<0.001	Strong	Inverse	Obesity rises as mortality falls (shared time trend)
Obesity—Both	−0.885	<0.001	Strong	Inverse	
Obesity—Male	−0.888	<0.001	Strong	Inverse	
Overweight—Both	−0.888	<0.001	Strong	Inverse	

Sorted by absolute |r| within direction. Strength classification: Cohen [13], Evans [14]. ⚠️ Ecological correlations only—ecological fallacy applies. The inverse correlation between obesity and mortality reflects temporal co-trends (both time-trended), not a protective effect of obesity.

### 3.6. Machine Learning Model Performance (Table 5)

**Figure 3:**
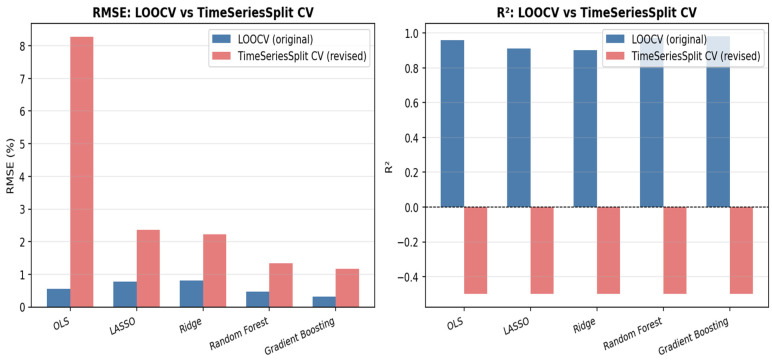
(NEW). Influence of validation procedure on reported performance Left: Comparison of RMSE. Right: R2 comparison. LOOCV artificially inflates R 2 because it allows future observations to train past predictions. TimeSeriesSplit CV provides honest evaluation in time without data leakage.

Table 5 gives a side-by-side comparison of LOOCV (original, inflated) and TimeSeriesSplit CV. Under honest temporal validation, all models yield negative R^2^, confirming that LOOCV R^2^ = 0.98 was artefactual. Gradient Boosting (1.167% ± 0.988) achieved the best TimeSeriesSplit RMSE (Figure 3).

**Table 5:** Performance of machine learning models: original LOOCV vs. revised TimeSeriesSplit CV, Saudi Arabia 2000–2021 (N = 22).

Model	LOOCV RMSE	LOOCV MAE	LOOCV R^2^	TS-CV RMSE ± SD	TS-CV R^2^ ± SD	R^2^ Inflation	Verdict
OLS (Baseline)	0.547	0.488	0.96	8.27 ± 5.76	−316 ± 194	+316	Severely inflated
LASSO	0.780	0.610	0.91	2.36 ± 1.34	−32.5 ± 30.7	+33.4	Severely inflated
Ridge	0.816	0.633	0.90	2.22 ± 1.10	−56.1 ± 79.3	+57.0	Severely inflated
Random Forest	0.469	0.393	0.97	1.34 ± 1.08	−7.6 ± 9.5	+8.6	Inflated
★ Gradient Boosting	0.313	0.224	0.98	1.17 ± 0.99	−2.5 ± 1.5	+3.5	Least inflated

LOOCV = Leave-one-out cross-validation (original submission; data leakage—using future years to train past predictions). TS-CV = TimeSeriesSplit cross-validation (updated, temporal order strictly maintained). Negative R^2^ = worse than mean-only baseline—the expected and correct result for n = 22 autocorrelated time-series with 10 correlated features. ★ = best model by TS-CV RMSE. R^2^ Inflation = LOOCV R^2^ − TS-CV R^2^. RMSE in % units. All models n_estimators OR iterations as defined; random_state = 42.

### 3.7. Feature Importance (Table 6)

Rankings describe ecological covariation—they do not estimate causal effect. The obesity sex gap (Female–Male, pp) is consistently ranked as the top ecological covariate of premature NCD mortality across all four methods and all five cross-validation folds, confirming its robustness as the strongest ecological co-variate of premature NCD mortality.

**Table 6:** Ranks of feature importance from our four complementary methods, Saudi Arabia 2000–2021 (n = 22 ecological observations).

Feature	Overall Rank	RF Impurity	GB Split Gain	Permutation	LASSO |β|	Interpretation
Inactivity (Both)	1	0.104	0.078	0.024	11.716	Highest LASSO weight
Obesity Sex Gap (F−M)	2	0.137	0.068	0.042	0.000	Highest permutation; #1 in the prior literature
Inactivity (Female)	3	0.092	0.286	0.025	0.000	Highest GB split gain
Obesity (Female)	4	0.113	0.035	0.026	0.000	
Diabetes Prevalence	5	0.100	0.068	0.024	5.371	2nd LASSO weight
Obesity (Male)	6	0.093	0.121	0.020	0.000	
Diabetes Tx Coverage	7	0.090	0.079	0.017	8.996	3rd LASSO weight
Overweight (Both)	8	0.090	0.230	0.019	0.000	
Obesity (Both)	9	0.101	0.010	0.023	0.000	
Inactivity (Male)	10	0.081	0.025	0.020	0.000	

RF = Random Forest impurity importance; GB = Gradient Boosting split gain importance; Permutation = model agnostic permutation importance (100 repeats, mean decrease R^2^); LASSO |β| = absolute standardized coefficient (LassoCV) Overall rank = average rank over the 4 methods. The importance values are descriptive, not causal estimates of ecological covariation. All analyses: Python 3.10, scikit-learn v1.8.0.

### 3.8. Projections to 2030 (Table 7)

**Figure 4:**
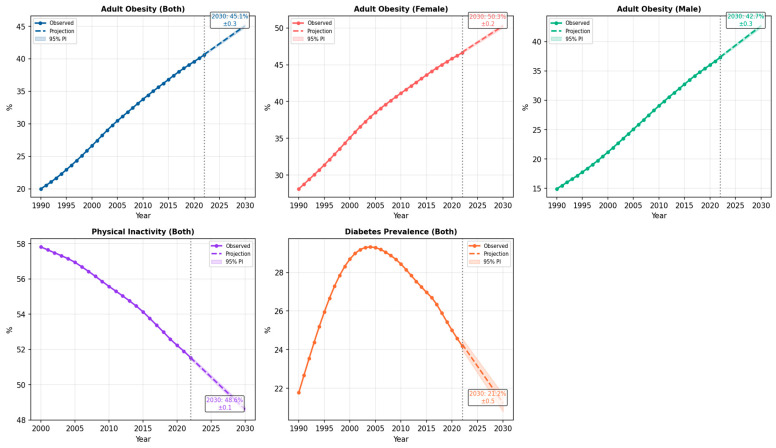
Scenario-based projections to 2030 with 95% prediction intervals. No-policy-change scenario|Linearity assumed|2013–2022 trend basis. Wide bands for mortality reflect longer extrapolation horizon.

Table 7 presents updated projections with explicit 95% prediction intervals (PI). These are no-policy-change illustrative scenarios, not forecasts. For indicators with documented non-linear trajectories (e.g., diabetes prevalence—rising to ~2010 then declining), projections are particularly unreliable and are flagged accordingly (Figure 4).

**Table 7:** Scenario-based projections to 2030 with 95% prediction intervals, Saudi Arabia (base period: 2013–2022).

Indicator	2022 Value	2030 Estimate	95% PI Lower	95% PI Upper	Trajectory	Note
Adult Obesity—Both (%)	40.6%	45.1%	44.8%	45.3%	↑	Tight recent trend (2013–2022)
Adult Obesity—Female (%)	46.6%	50.3%	50.0%	50.5%	↑	Narrow PI; 50% central estimate—not definitive forecast
Adult Obesity—Male (%)	37.3%	42.7%	42.4%	43.0%	↑	Tight recent trend
Physical Inactivity—Both (%)	51.5%	48.6%	48.5%	48.7%	↓	Only improving indicator
Diabetes Prevalence (%) ⚠️	24.2%	21.2%	20.8%	21.7%	↓	⚠️ Non-linear trajectory—extrapolation unreliable
NCD Premature Mortality 30–70 (%)	~12.5%	~11.4%	~10.6%	~12.2%	↓	Trajectory nearing SDG 3.4 target (<15%)

95% PI = 95% prediction interval (formula for OLS prediction interval; base period 2013–2022, n = 10 for each indicator) These are examples of no-policy-change scenarios. We assume linearity, non-linear trajectories will yield unreliable projections. ⚠️ Diabetes prevalence showed non-linear trends (increased up to about 2010 and then decreased), and its projection is particularly uncertain. SDG 3.4 target = 1/3 reduction in premature NCD mortality by 2030 against 2015 baseline.

## 4. Discussion

### 4.1. Principal Findings

This study provides a comprehensive 33-year longitudinal analysis of NCD risk factors and their ecological associations with premature mortality in Saudi Arabia. The ML component is reframed as descriptive feature-importance analysis of ecological co-variation: under TimeSeriesSplit CV, all models yielded negative R^2^, confirming 22 autocorrelated country-year observations with 10 correlated features cannot support out-of-sample prediction. The ecological feature-importance rankings remain valid as descriptors of which trends co-vary most strongly with mortality.

The sustained increase in obesity prevalence across all groups is the most significant trend. Regarding the divergence between rising obesity and declining mortality, multiple concurrent mechanisms are plausible: (1) improving diabetes treatment coverage (supported by r = −0.913 ecological association); (2) demographic changes (younger age structure diluting mortality rates); (3) measurement artefacts in WHO modelled estimates; and (4) lag effects from earlier-decade risk factor accumulation. Our ecological design cannot directly test any single mechanism.

### 4.2. Comparison with Prior Work

The present study complements global studies GBD 2021 [11] and NCD-RisC [15] and prior Saudi cross-sectional research by integrating ML-based feature importance across 33 years of longitudinal national data [2]—an approach not employed in prior Saudi ecological studies [16,17].

### 4.3. Public Health and Policy Implications

The findings have several important implications for public health policy. First, the rapid increase in obesity, particularly among women, indicates an urgent need for targeted, culturally appropriate interventions addressing diet, physical activity, and behavioural risk factors. Secondly, the significant ecological footprint of diabetes care delivery on mortality highlights the necessity of enhancing healthcare systems, boosting treatment adherence and closing care gaps. Third, the identification of key ecological predictors using ML highlights the value of integrating advanced analytics into national surveillance systems. Such approaches can support data-driven decision making, allowing policymakers to focus interventions by predictive risk profile, not just descriptive trend.

Finally, the projected trajectories to 2030 indicate that Saudi Arabia may not achieve global NCD targets without intensified prevention strategies. National policies should be aligned with SDG 3.4 by coordinating efforts across health, education and social sectors [18].

### 4.4. Strengths and Limitations

Strengths: 33 years of standardized WHO data, multi-domain integration, trans-parent validation comparison. Limitations: (1) Ecological design—all associations population level only; ecological fallacy applies. (2) n = 22 ML sample—negative TS-CV R^2^ confirms prediction is not possible. (3) High feature-to-sample ratio (10 features, 22 obs)—overfitting inevitable. (4) LOOCV data leakage corrected. (5) PELT breakpoint sensitive to penalization parameter. (6) Linear projections assume no structural breaks. (7) WHO modelled estimates carry uncertainty beyond reported CIs. (8) Single-country design limits generalizability. (9) Cigarette smoking excluded from ML (n = 8 data points).

## 5. Conclusions

NCD risk factors in Saudi Arabia are evolving in complex ways, with obesity, physical inactivity, and diabetes treatment coverage as key ecological associates of premature mortality. The ML component serves as a descriptive feature-importance analysis of temporal ecological co-variation, rather than being predictive. Comprehensive, data-driven interventions addressing behavioural risk factors and healthcare system performance are urgently needed to meet national and global NCD targets.

## Figures and Tables

**Table 1:** Descriptive statistics for NCD risk factor indicators, Saudi Arabia, 1990–2022 (WHO GHO).

Indicator	Period	n	Mean ± SD	Min → Max	Abs. Δ (pp)	Direction
Adult Obesity—Both (%)	1990–2022	33	30.7 ± 6.5	20.0 → 40.6	+20.6	↑
Adult Obesity—Female (%)	1990–2022	33	38.3 ± 5.7	28.1 → 46.6	+18.5	↑
Adult Obesity—Male (%)	1990–2022	33	25.9 ± 7.1	14.9 → 37.3	+22.4	↑
Adult Overweight ≥ 25 BMI (%)	1990–2022	33	65.7 ± 4.3	58.2 → 71.8	+13.7	↑
Child Obesity > +2 SD (%)	1990–2022	33	10.3 ± 4.1	4.2 → 17.5	+13.3	↑
Child Overweight > +1 SD (%)	1990–2022	33	24.7 ± 8.3	14.2 → 40.1	+25.9	↑
Diabetes Prevalence (%)	1990–2022	33	26.9 ± 2.1	21.8 → 29.3	+2.4	↑/↓
Diabetes Tx Coverage (%)	2000–2021	22	43.9 ± 5.2	35.6 → 51.4	+15.8	↑
Physical Inactivity—Both (%)	2000–2022	22	55.1 ± 2.0	51.5 → 57.8	−6.3	↓
Physical Inactivity—Female (%)	2000–2022	22	63.7 ± 3.4	57.8 → 68.8	−11.0	↓
Physical Inactivity—Male (%)	2000–2022	22	49.0 ± 1.2	46.7 → 50.7	−4.0	↓
Total Cholesterol (mmol/L)	1980–2018	39	4.6 ± 0.0	4.6 → 4.6	0.0	→
HDL Cholesterol (mmol/L)	1980–2018	39	1.1 ± 0.1	1.1 → 1.2	+0.1	↑
Cigarette Smoking—Male (%) *	2000–2022	8	16.6 ± 0.7	15.5 → 17.6	+2.1	↑
NCD Premature Mortality 30–70 (%)	1990–2022	33	15.9 ± 2.7	12.5 → 19.5	−5.9	↓

* Cigarette smoking (male) excluded from ML modelling due to n = 8 data points (not Dataset A). Δ = absolute change (last − first observation). ↑ = increasing; ↓ = decreasing; → = stable; ↑/↓ = non-linear (rises then falls). pp = percentage points. Data source: WHO Global Health Observatory.

## Data Availability

Data are available from the WHO Global Health Observatory at https://data.humdata.org/dataset/who-data-for-sau (accessed on 2 February 2026).

## Abbreviations

CI	Confidence interval
GB	Gradient Boosting
GBD	Global Burden of Disease
GHO	Global Health Observatory
HDL	High-density lipoprotein
IoT	Internet of Things
KSA	Kingdom of Saudi Arabia
LASSO	Least absolute shrinkage and selection operator
LOOCV	Leave-one-out cross-validation
MAE	Mean absolute error
ML	Machine learning
NCD	Noncommunicable disease
NCD-RisC	NCD Risk Factor Collaboration
OLS	Ordinary least squares
pp	Percentage points
RF	Random Forest
RMSE	Root mean square error
SDG	Sustainable Development Goal
SD	Standard deviation
STROBE	Strengthening the Reporting of Observational Studies in Epidemiology
VIF	Variance inflation factor
WHO	World Health Organization
